# The Naval Medical Service

**Published:** 1899-09-16

**Authors:** 


### The Hospital

A Journal of

The Medical Sciences and Hospital Administration.

Vol. XXVI.—No. 677.] Edited by Sir Henry Burdett, K.C.B., and Solomon C. Smith, M.D., M.R.C.P. [September 16, 1899.

### The Naval Medical Service.

It is eminently satisfactory to find that, while the strength, efficiency, and manning of the Navy have of late years attracted a considerable amount of critical attention, insomuch that the several departments concerned have been compelled by public opinion to abandon their ordinary attitude of official optimism, and to bestir themselves in various directions, the very important medical department will not be suffered to lag behind in the race of reform, but has every prospect of being brought, before long, into a position corresponding to recent advances in knowledge; so that naval surgeons will be able to afford to the officers and men under their care the same advantages that these could derive from treatment in a well-organised modern hospital. At the present time, although naval surgeons have always been better treated by the authorities than their brethren in the sister service, the regulations under which their duties are performed are not of a kind calculated to promote the highest attainable efficiency; and, when this was brought home to the perceptions of those most interested, a Departmental Committee was appointed by the Admiralty to inquire into certain specific questions bearing upon the subject. These were: (1) The system of entry and training of naval medical officers, with special reference to the sufficiency of the Haslar course of instruction for the fulfilment of the purposes for which it was devised; and (2) the facilities afforded to naval medical officers for the renewal or increase of their professional knowledge. The Committee consisted of Rear-Admiral A. W. Moore, C.B., Mr. Austen Chamberlain, M.P., Sir Henry F. Norbury, the Medical Director-General, and Staff-Surgeon Gipps, R.N. Their report was issued last week as a Parliamentary paper, and it will be found to contain recommendations which, if acted upon (as they surely will be), cannot fail to be of great advantage to the service.

The Committee recommend that the course of instruction now given at Haslar should be extended from three months to four, and in many respects remodelled, the subjects of meteorology and of food analysis being much curtailed, in order to make way for more thorough instruction in the diagnosis and treatment of diseases peculiar to foreign stations. This is at present better given at Netley than at Haslar and the recommendation is practically that the two services should be placed upon a level in this respect. The possibility of using for the purpose the new School of Tropical Medicine which is being established in connection with the Royal Albert Dock Hospital was considered by the Committee, who decided that, except to a very limited extent (as a centre for a post-graduate course), this school and hospital could not be made available for naval purposes. It was thought better that the required instruction should be given at Haslar, and that special wards should be set apart there for the treatment of the diseases in question. This decision was probably the wisest that could have been formed, because its effect will be to render the naval medical service self-contained and independent, and to secure that the necessary special instruction shall be imparted by the superior officers of those who are to receive it. It is further proposed that the seniority of naval medical officers shall be made to depend upon the combined results of the competitive examination by which they enter the service, and of a leaving examination at the conclusion of the Haslar course, instead of, as at present, upon the former alone, so as to offer a reward for diligence in the special studies which they will be called upon to pursue. The present system, by which every year seven naval surgeons are allowed three months' leave on full pay, for the purpose of professional study, is pronounced by the Committee to be inadequate to the requirements of the case; and it is recommended that the number of officers thus privileged should be increased, and that they should be taken from all ranks below that of Deputy-Inspector-General. The new Haslar course, in addition to instruction in the diseases of foreign stations, is to include bacteriology, especially in its bearing upon the above diseases, examination of blood for diagnostic purposes, serum-therapeutics, including that of snake bites, naval surgery in relation to bullet wounds, and the use of the Röntgen ray apparatus. It is further proposed to remodel the present form of the "Annual Health Report of the Navy," with a view to diminish the drudgery now cast upon officers in preparing the necessary returns; and this change will admit of its being rendered more available as a means of publishing valuable contributions to medicine, surgery, and pathology, such as the special opportunities of the service would often allow the officers to provide. Another recommendation which will be extremely acceptable is that the present absurd regulation, under which naval surgeons are compelled to find their own instruments, shall be rescinded, and that all instruments not of a personal kind, such as stethoscopes, thermometers, and pocket cases, shall be supplied to each ship when she is commissioned, and shall remain the property of the public. The present "regulation" stock of instruments costs each officer from £50 to £60, and is necessarily never abreast of the constantly increasing requirements of practice.

